# No-show Prediction Model Performance Among People With HIV: External Validation Study

**DOI:** 10.2196/43277

**Published:** 2023-03-29

**Authors:** Joseph A Mason, Eleanor E Friedman, Juan C Rojas, Jessica P Ridgway

**Affiliations:** 1 The Chicago Center for HIV Elimination Department of Medicine University of Chicago Chicago, IL United States; 2 Division of Pulmonary & Critical Care Medicine Department of Medicine Rush University Medical Center Chicago, IL United States

**Keywords:** no-show, prediction model, Epic systems, people with HIV, human immunodeficiency virus, electronic medical record, external validation, technology, model, care, patient, HIV

## Abstract

**Background:**

Regular medical care is important for people living with HIV. A no-show predictive model among people with HIV could improve clinical care by allowing providers to proactively engage patients at high risk of missing appointments. Epic, a major provider of electronic medical record systems, created a model that predicts a patient’s probability of being a no-show for an outpatient health care appointment; however, this model has not been externally validated in people with HIV.

**Objective:**

We examined the performance of Epic’s no-show model among people with HIV at an academic medical center and assessed whether the performance was impacted by the addition of demographic and HIV clinical information.

**Methods:**

We obtained encounter data from all in-person appointments among people with HIV from January 21 to March 30, 2022, at the University of Chicago Medicine. We compared the predicted no-show probability at the time of the encounter to the actual outcome of these appointments. We also examined the performance of the Epic model among people with HIV for only HIV care appointments in the infectious diseases department. We further compared the no-show model among people with HIV for HIV care appointments to an alternate random forest model we created using a subset of seven readily accessible features used in the Epic model and four additional features related to HIV clinical care or demographics.

**Results:**

We identified 674 people with HIV who contributed 1406 total scheduled in-person appointments during the study period. Of those, we identified 331 people with HIV who contributed 440 HIV care appointments. The performance of the Epic model among people with HIV for all appointments in any outpatient clinic had an area under the receiver operating characteristic curve (AUC) of 0.65 (95% CI 0.63-0.66) and for only HIV care appointments had an AUC of 0.63 (95% CI 0.59-0.67). The alternate model we created for people with HIV attending HIV care appointments had an AUC of 0.78 (95% CI 0.75-0.82), a significant improvement over the Epic model restricted to HIV care appointments (*P*<.001). Features identified as important in the alternate model included lead time, appointment length, HIV viral load >200 copies per mL, lower CD4 T cell counts (both 50 to <200 cells/mm^3^ and 200 to <350 cells/mm^3^), and female sex.

**Conclusions:**

For both models among people with HIV, performance was significantly lower than reported by Epic. The improvement in the performance of the alternate model over the proprietary Epic model demonstrates that, among people with HIV, the inclusion of demographic information may enhance the prediction of appointment attendance. The alternate model further reveals that the prediction of appointment attendance in people with HIV can be improved by using HIV clinical information such as CD4 count and HIV viral load test results as features in the model.

## Introduction

Epic Systems is a major provider of health information technology in the United States, providing electronic medical records for more than 250 million patients [1]. Epic’s platform includes predictive models for patient care, including a model that predicts a patient’s probability of being a no-show for an outpatient health care appointment. Epic reports the area under the receiver operating characteristic curve (AUC) of this model to range between 0.71 to 0.81, but the model has not been externally validated in certain groups of patients, including people with HIV [2]. Previous external validation of Epic’s proprietary sepsis prediction model showed reduced performance [3].

Regular medical care for people with HIV is of the utmost importance [4,5]. Missed medical appointments among people with HIV are independently associated with increased mortality [6]. Additionally, HIV is a disease of extreme disparity, with persons of racial and sexual minorities disproportionately impacted [7]. A no-show predictive model among people with HIV could improve clinical care by allowing providers to proactively engage patients at high risk of missing appointments. We examined the performance of Epic’s no-show model among people with HIV at an academic medical center and whether the performance was impacted by the addition of demographic and HIV clinical information.

## Methods

### Overview

On January 21, 2022, the University of Chicago Medicine (UCM) Epic system implemented the second version of the Epic no-show predictive model. The second version of this model is a random forest using 22 features developed using data from 600,000 appointments from February to October 2020 from two health care organizations [2]. The 600,000 appointments used to train the model were not limited to HIV-related appointments. The health care organizations that contributed data for the creation of this model were not identified by Epic [2]. Features included variables related to past appointment history and appointment characteristics. The model output is the predicted probability of each patient being a no-show for each appointment.

To measure the performance of the Epic model among people with HIV, we used an existing registry of people with HIV at UCM. We then obtained encounter data from all in-person appointments among people with HIV from January 21 to March 30, 2022, and compared the predicted no-show probability at the time of the encounter to the actual outcome of these appointments. We also examined the performance of the Epic model among people with HIV for only HIV care appointments in the infectious diseases department.

We further compared the no-show model among people with HIV for HIV care appointments to an alternate random forest model we created using a subset of seven readily accessible features used in the Epic model and four additional features related to HIV clinical care or demographics (Table 1). Additional features added to the model included viral load (<200 vs ≥200 copies/mL) and CD4 T cell count (<50, <200, <350, ≥350 cells/mm^3^) as well as race (Black or African American, White, more than one race, patient declined, and unknown/not reported) and sex at birth (male and female). Features were either continuous (appointment lead time), binary (referral to appointment), or categorical (race).

For HIV care appointment models, data were randomly split into training (70%) and testing (30%) data sets to standardize the comparison. Model performance was evaluated using AUC via DeLong et al’s [8] method. Feature importance in the model was evaluated using scaled Gini index scores, with higher scaled Gini index scores indicating features with a greater impact on model prediction [9].

**Table 1:** No-show prediction model and clinical features for people with HIV at the University of Chicago Medicine, January 21 to March 30, 2022.

Model	Patients, n	Rows, n	Features, n	Clinical features
Epic, people with HIV, all appointments model	674	9100	22	Appointment changed, appointment day of the week, appointment hour of day, appointment month of year, appointment zip code, confirmation status, department ID, department specialty, last communication, appointment lead time, patient called, referral to appointment, referral to appointment required, rescheduled appointment, service area ID, visit type, no-show rate, number of past appointments, number of past canceled appointments, number of past ED^a^ visits, number of past hospitalizations, number of scheduled appointments
Epic, people with HIV, HIV care appointments model	331	2835	22	Appointment changed, appointment day of the week, appointment hour of day, appointment month of year, appointment zip code, confirmation status, department ID, department specialty, last communication, appointment lead time, patient called, referral to appointment, referral to appointment required, rescheduled appointment, service area ID, visit type, no-show rate, number of past appointments, number of past canceled appointments, number of past ED visits, number of past hospitalizations, number of scheduled appointments
Alternate, people with HIV, HIV care appointments model	331	2835	11	Department ID, department specialty, appointment lead time, referral to appointment, appointment rescheduled, visit type, appointment changed, viral load categories, CD4 categories, race, sex

^a^ED: emergency department.

### Ethical Considerations

This project underwent a formal review and received a determination of quality improvement according to UCM institutional policy. Thus, this initiative was not considered human subjects research and was not reviewed by the institutional review board. All UCM patient data were deidentified prior to use.

## Results

**Figure 1:**
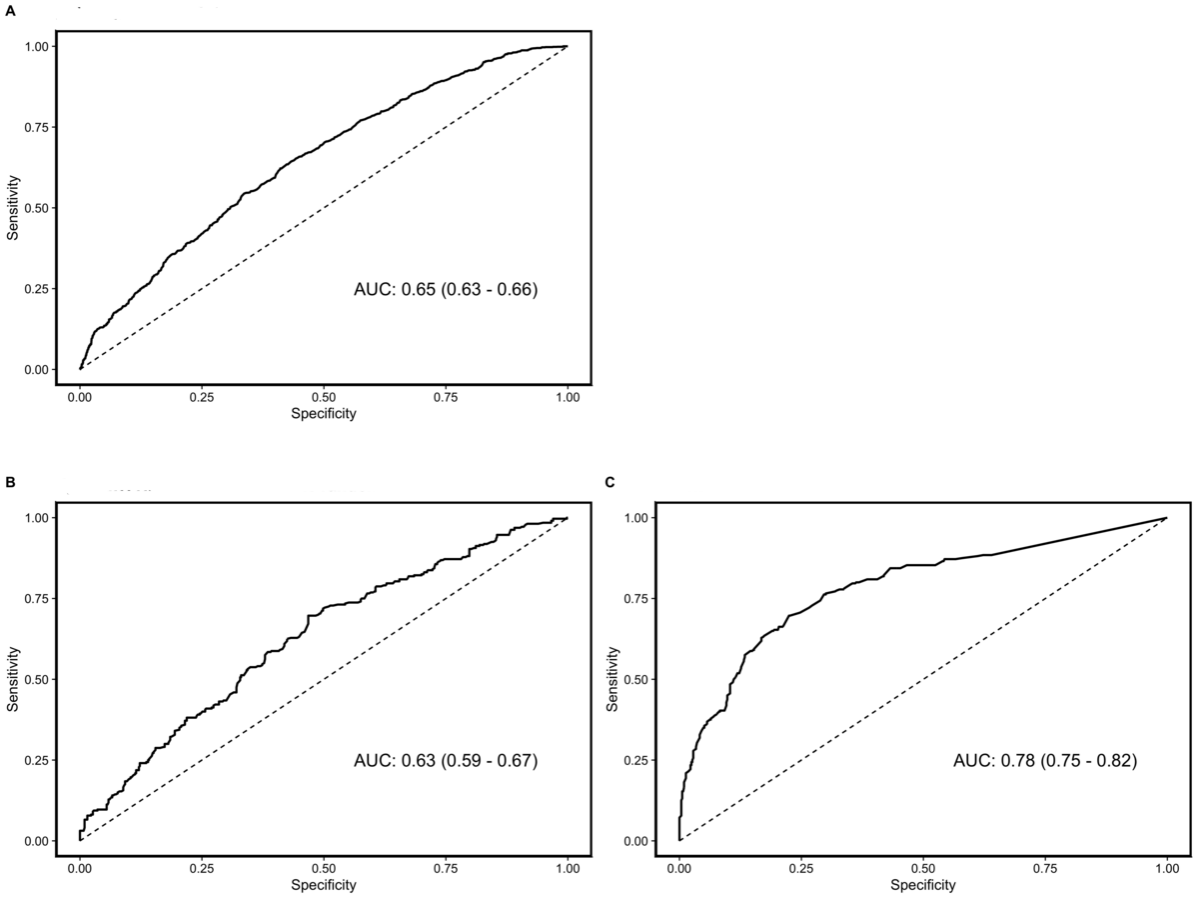
No-show prediction model receiver operating characteristic curves for PwH at the University of Chicago Medicine, January 21 to March 30, 2022. (A) Epic PwH all appointments random forest model. (B) Epic PwH HIV care appointments random forest model. (C) Alternate PwH HIV appointments random forest model. AUC: area under the receiver operating characteristic curve; PwH: people with HIV.

We identified 674 people with HIV who contributed 1406 total scheduled in-person appointments during the study period. The performance of the Epic model among people with HIV for all appointments in any outpatient clinic had an AUC of 0.65 (95% CI 0.63-0.66). When we restricted the data to include only HIV care clinic appointments, we identified 331 people with HIV who contributed 440 infectious disease appointments. The AUC of the Epic model for HIV care appointments among people with HIV was 0.63 (95% CI 0.59-0.67), and there was no significant difference in performance compared to the model that included all appointments (*P*=.36). The alternate model we created for people with HIV attending HIV care appointments had an AUC of 0.78 (95% CI 0.75-0.82), a significant improvement over the Epic model restricted to HIV care appointments (*P*<.001; Figure 1). Features identified as important in this model included ones from the original Epic model, such as appointment lead time and appointment length. However, some of the new demographic and clinical features were also identified as important, such as viral load >200 copies per mL, lower CD4 T cell counts (both 50 to <200 cells/mm^3^ and 200 to <350 cells/mm^3^), and female sex.

## Discussion

We examined the performance of the Epic no-show predictive model among people with HIV using all appointments regardless of specialty and among HIV care appointments at the infectious disease clinic. For both models among people with HIV, performance was significantly lower than reported by Epic.

We found that a model that incorporated a subset of the features used in the original Epic model along with demographic and HIV clinical information performed better among people with HIV attending HIV care appointments. Similar to previous studies, our model showed that HIV clinical information (CD4 count and HIV viral load laboratory test results) are important for predicting future appointment attendance in people with HIV [10,11]. The inclusion of demographic factors further improved model performance.

A limitation of this project was our inability to fully replicate all 22 features from Epic’s no-show prediction model due to the proprietary nature of the model. We were able to identify seven features used by Epic and include these as well as additional clinical and demographic features in the alternate model to see if the additional features would improve the model performance among people with HIV. Two of the model features (race and sex) added in our alternate model were originally part of version 1 of the Epic no-show model but were later removed due to concerns about adverse impacts on marginalized groups [2].

The improvement in the performance of the alternate model over the proprietary Epic model demonstrates that among populations with extreme disparities, such as people with HIV, the inclusion of demographic information may enhance the prediction of appointment attendance. The alternate model further reveals that the prediction of appointment attendance in people with HIV can be improved by using HIV clinical information such as CD4 count and HIV viral load test results as features in the model. Hospitals and electronic medical record systems interested in developing or improving their no-show prediction models among diseases with high patient disparity may benefit from including these features.

## Abbreviations

AUC: area under the receiver operating characteristic curve
UCM: University of Chicago Medicine

## Data Availability

The data sets generated or analyzed during this study are not publicly available due to patient privacy but are available from the corresponding author upon reasonable request.
